# Avian Influenza H5N1 in Tigers and Leopards

**DOI:** 10.3201/eid1012.040759

**Published:** 2004-12

**Authors:** Juthatip Keawcharoen, Kanisak Oraveerakul, Thijs Kuiken, Ron A.M. Fouchier, Alongkorn Amonsin, Sunchai Payungporn, Suwanna Noppornpanth, Sumitra Wattanodorn, Apiradee Theamboonlers, Rachod Tantilertcharoen, Rattapan Pattanarangsan, Nlin Arya, Parntep Ratanakorn, Albert D.M.E. Osterhaus, Yong Poovorawan

**Affiliations:** * Chulalongkorn University, Bangkok, Thailand;; † Erasmus Medical Centre, Rotterdam, the Netherlands;; ‡ Mahidol University, Salaya, Nakorn Pathom, Thailand

## Abstract

Influenza virus is not known to affect wild felids. We demonstrate that avian influenza A (H5N1) virus caused severe pneumonia in tigers and leopards that fed on infected poultry carcasses. This finding extends the host range of influenza virus and has implications for influenza virus epidemiology and wildlife conservation.

## The Study

The 2003–2004 avian influenza A (H5N1) virus outbreak in Southeast Asia resulted in 24 reports of fatal human cases (May 12, 2004) due to direct transmission of the virus from birds to humans. During the H5N1 virus outbreak in Thailand in December 2003 (*1*) , two tigers (*Panthera tigris*) and two leopards (*P. pardus*) at a zoo in Suphanburi, Thailand, showed clinical signs, including high fever and respiratory distress, and they died unexpectedly. The animals had been fed fresh chicken carcasses from a local slaughterhouse. At that time many chickens around Suphanburi were dying with respiratory and neurologic symptoms of what was retrospectively identified as H5N1 virus infection (*1*). Postmortem examinations were performed on all four zoo felids, and samples were collected for histologic, immunohistochemical, and virologic analyses.

**Figure 1:**
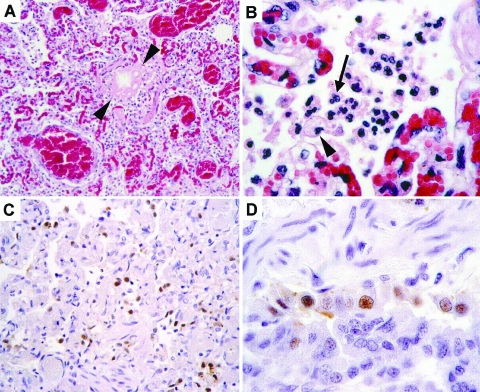
Histopathologic and immunohistochemical evidence of avian influenza A (H5N1) virus in leopard lung. A) Diffuse alveolar damage in the lung: alveoli and bronchioles (between arrowheads) are flooded with edema fluid and inflammatory cells. B) Inflammatory cells in alveolar lumen consist of alveolar macrophages (arrowhead) and neutrophils (arrow). C) Many cells in affected lung tissue express influenza virus antigen, visible as brown staining. D) Expression of influenza virus antigen in a bronchiole is visible mainly in nuclei of epithelial cells.

At necropsy, the primary gross lesions in all four animals were severe pulmonary consolidation and multifocal hemorrhage in several organs, including lung, heart, thymus, stomach, intestine, liver, and lymph nodes. Histologic examination was performed on formalin-fixed, paraffin-embedded tissue sections stained with hematoxylin and eosin. Pulmonary lesions were characterized by loss of bronchiolar and alveolar epithelium; thickening of alveolar walls; and flooding of alveolar lumens with edema fluid mixed with fibrin, erythrocytes, neutrophils, and macrophages (Figure 1, A and 1B). One tiger and one leopard had evidence of encephalitis, characterized by multifocal infiltration by neutrophils and macrophages. Tissues were examined for influenza A (H5N1) virus nucleic acid by reverse transcriptase–polymerase chain reaction (RT-PCR) analysis, with primer pairs specific for the hemagglutinin (HA) and neuraminidase (NA) genes (*2*). Lung samples from all four animals were positive for H5N1 with both primer pairs, and the identity of the PCR products was confirmed by nucleotide sequencing. Formalin-fixed, paraffin-embedded tissue sections from one of the leopards were examined for influenza virus antigen by a immunohistochemical technique (*3*). A monoclonal antibody against the nucleoprotein of influenza A virus was used as primary antibody. Alveolar and bronchiolar epithelial cells in affected lungs expressed influenza virus antigen (Figure 1, C and 1D), confirming that influenza virus infection was the primary cause of the pneumonia.

**Figure 2:**
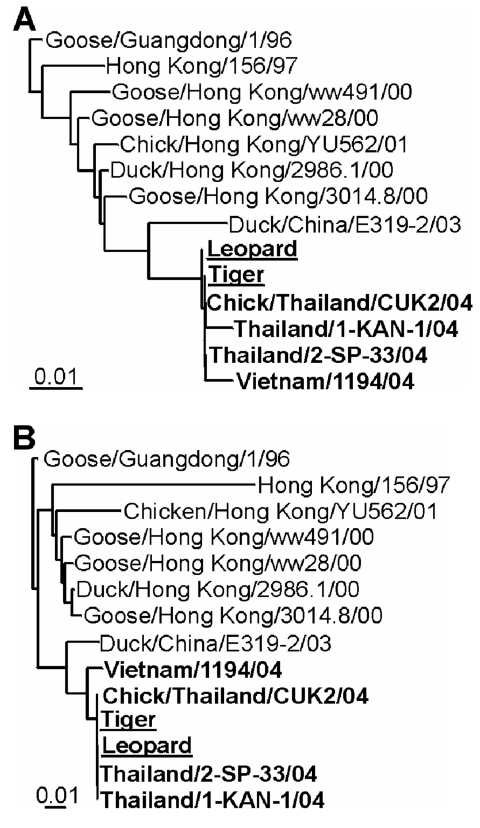
Phylogenetic comparison of zoo felid isolates with other H5N1 viruses. DNA maximum likelihood tree of hemagglutinin and neuraminidase sequences. Representative full-length Asian influenza A virus H5 (A) and N1 (B) sequences from 1996 to 2004 are shown with 2004 sequences in bold and leopard and tiger sequences underlined. Maximum likelihood trees were generated by using 100 bootstraps and three jumbles, and the resulting consensus trees were used as a user tree to recalculate branch lengths. The trees had good bootstrap support. Scale bars roughly indicate 1% nucleotide difference between related strains. Accession no. used: A/Goose/Guangdong/1/1996 (AF144305 and AF144304), A/Hong Kong/156/1997 (AF028709 and AF028708), A/Goose/Hong Kong/ww491/2000 (AY059480 and AY059489), A/Goose/Hong Kong/ww28/2000 (AY059475 and AY059484), A/Chicken/Hong Kong/YU562/2001 (AY221529 and AY221547), A/Duck/Hong Kong/2986.1/2000 (AY059481 and AY059490), A/Goose/Hong Kong/3014.8/2000 (AY059482 and AY059491), A/duck/China/E319-2/2003 (AY518362 and AY518363), A/Thailand/1-KAN-1/2004 (AY555150 and AY555151), A/Thailand/2-SP-33/2004 (AY555153 and AY555152), A/Chicken/Thailand/CU-K2/2004 (AY590568 and AY590567), A/Leopard/Thailand/2004 (AY646175 and AY646176), and A/Tiger/Thailand/2004 (AY646167 and AY646168).

Influenza A virus was isolated from lung samples of one of the tigers and one of the leopards by injecting into embryonated chicken eggs (*3*). The entire genomes of these two viruses were sequenced. RT-PCR specific for the conserved noncoding regions of influenza A virus was performed (*4*). PCR products were purified by using the QIAquick gel extraction kit (Qiagen, Leusden, the Netherlands) and sequenced with the Big Dye Terminator sequencing kit, version 3.0 (Amersham Biosciences, Piscataway, NJ). Nucleotide sequences were aligned by using Clustal-W running under BIOEDIT 5.0.9 (Ibis Therapeutics, Carlsbad, CA) and maximum likelihood trees were generated with PHYLIP 3.6 (University of Washington, Seattle, WA) (*5*) with 100 bootstraps and three jumbles. The consensus tree was used as a user tree in DNAML to recalculate branch lengths. The trees had good bootstrap support (data not shown). Sequencing and phylogenetic analysis of the HA and NA genes of these two isolates showed that they were virtually identical to each other and to the H5N1 virus circulating in poultry at the time (Figure 2) (*6*). Therefore, the zoo felids were most probably directly infected with avian influenza A (H5N1) virus by feeding on infected poultry carcasses. Furthermore, phylogenetic analysis of the remaining six genome segments (data not shown; leopard accession no. AY646177–AY646182; tiger accession no. AY646169–AY646174) showed that they were of avian origin, which indicates that no reassortment with mammalian influenza viruses had occurred.

The virus isolates obtained from the tiger and the leopard contained a glutamine at position 222 (226 in H3) and a glycine at position 224 (228 in H3) in HA1, which were also found in other recent H5N1 isolates and which are related to preferential binding to avian cell-surface receptors (*7*). Both viruses contained a deletion of five amino acid residues in NS1, like other recent H5N1 isolates, and contained a glutamic acid at position 92 (*6**,**8*). The mutation glutamic acid to lysine at position 627 of PB2, which was responsible for the high virulence of A/Hong Kong/483/97 and was also found in fatal human cases of H7N7 infection in the Netherlands, was observed in the virus isolate obtained from the leopard, but not from the tiger (*9**,**10*). Thus, with the exception of position 627 at PB2 in the leopard isolate, the genomic sequences of these zoo felid isolates did not show substantial differences from other recent H5N1 isolates from Asia.

Lung samples from all four felids tested negative for canine distemper virus by RT-PCR (*11*), while those of three of four felids tested positive for a vaccine strain of feline panleukopenia virus (*12*), administered 2 weeks before death. Although absence of typical clinical signs and lesions ruled out feline panleukopenia as the primary cause of death, an immunosuppressive effect cannot be ruled out (*13*).

## Conclusions

This report is the first of influenza virus infection causing disease or death in nondomestic felids. Generally, influenza virus is also not considered pathogenic for the domestic cat. Experimental infection of domestic cats in the 1970s and 1980s with influenza A viruses of subtypes H3N2 from humans, H7N3 from a turkey, and H7N7 from a harbor seal (*Phoc vitulina*) resulted in transient virus excretion and a temporary increase in body temperature but did not induce clinical signs of disease (*14**–**16*). However, anecdotes of fatal infection have been reported in this species during the 2003–2004 H5N1 virus outbreak (*17*) , and these reports were recently confirmed experimentally (*18*).

Our findings in tigers and leopards extend the host range of this virus and, together with the findings in domestic cats (*18*), suggest that this H5N1 virus is more pathogenic for felids than other influenza viruses. This finding has important implications for wildlife conservation and influenza virus epidemiology. First, H5N1 virus infection may threaten the survival of endangered felids, as has been shown recently for other emerging viruses in susceptible wildlife (*19**,**20*). The severity of this threat is increased because H5N1 virus may be transmitted horizontally between domestic cats (*18*). Second, if the higher pathogenicity of H5N1 virus for felids also means longer excretion of more virus, the role of felids in avian influenza epidemiology, both in humans and in poultry, needs to be reevaluated. Finally, the confirmation of H5N1 virus infection as the probable cause of death in two other mammalian hosts besides humans implies that more species of mammals may be at risk for infection with this virus.
